# Anti-Inflammatory Activity of Rg3-Enriched Korean Red Ginseng Extract in Murine Model of Sepsis

**DOI:** 10.1155/2018/6874692

**Published:** 2018-10-11

**Authors:** Evelyn Saba, Dahye Jeong, Muhammad Irfan, Yuan Yee Lee, Sang-Joon Park, Chae-Kyu Park, Man Hee Rhee

**Affiliations:** ^1^Laboratory of Physiology and Cell Signaling, College of Veterinary Medicine, Kyungpook National University, Daegu 41566, Republic of Korea; ^2^Laboratory of Histology, College of Veterinary Medicine, Kyungpook National University, Daegu 41566, Republic of Korea; ^3^R&D Headquarters, Korean Ginseng cooperation, Daejeon 34520, Republic of Korea; ^4^Cardiovascular Research Institute, Kyungpook National University, Daegu 41944, Republic of Korea

## Abstract

Ginseng has therapeutic effects on various bodily disorders ranging from minor inflammation to major cardiovascular diseases. In our study, we explored the anti-inflammatory effects of Rg3-enriched red ginseng extract (Rg3-RGE), a ginsenoside belonging to the panaxadiol group. We employed nitric oxide assay (NO) and 3-(4,5-dimethylthiazol-2-yl)-2,5-diphenyltetrazolium bromide (MTT) assay, quantitative reverse transcriptase-polymerase chain reaction (qRT-PCR), western blot, and hematoxylin and eosin staining (H&E) to elucidate the anti-inflammatory activity of Rg3-RGE. Rg3-RGE potently suppressed NO production in the murine macrophage cell line, RAW 264.7 cells, without any cytotoxicity across dosages. Additionally, it inhibited the mRNA expression of proinflammatory mediators and cytokines like iNOS, COX-2, IL-1*β*, IL-6, and TNF-*α*. Moreover it also inhibited the levels of malondialdehyde levels in serum of septic shock mice. Immunoblot analysis showed that Rg3-RGE induced anti-inflammatory signal transduction via the NF-*κ*B and MAPK pathways. A remarkable attenuation of inflammation by oral treatment with Rg3-RGE in mice was observed in the survival study. The* in vivo* study using a septic shock mouse model also showed similar results as the* in vitro* study. Our findings suggest that Rg3-RGE can potentially be a potent anti-inflammatory agent that likely mediates its anti-inflammatory effects via the NF-*κ*B and MAPK pathways.

## 1. Introduction

Inflammation is an integrated response to a foreign invader that enters the body (among other stimuli). It includes a series of reactions and pathways by which the body, through its self-defense mechanisms, combats the invasion, and rids the body of the pathogen. However, an uncontrolled self-defense response towards a foreign invader can lead to excessive secretion of chemokines and cytokines that can damage the body. In such cases, the proper regulation of proinflammatory and anti-inflammatory agents is maintained either by the body itself or by exogenous sources such as anti-inflammatory drugs [1–3].

Panax ginseng has been used for medicinal purposes in the Korean Peninsula for centuries owing to its anti-inflammatory and antioxidant properties and other health-enhancing effects, such as increasing longevity, protecting the cardiovascular system, and boosting the immune system. Ginseng can be divided into two fractions on the basis of its chemical composition: saponins and nonsaponins. Both of these subclasses contain individual compounds such as Rg3, Rb1, Rb2, Rb3, and Rc. In particular, Rg3-enriched Korean red ginseng extract (RGE) has been studied extensively in the past for its vasodilating, anti-inflammatory, and antioxidant properties [4].

Particularly since the red ginseng fraction we used in our study is enriched in Rg3 so we would like to elucidate the effects of Rg3 as an antinociceptive because it modulates the Ca^2+^ channels (part of the pharmacologic basis of ginseng-mediated antinociception) and as an anti-inflammatory agent in the suppression of mouse ear swelling and prevention of vascular inflammatory diseases by decreasing the expression of intercellular adhesion molecule 1 (ICAM–1), vascular cell adhesion molecule-1 (VCAM-1), and P- and E-selectin [5–8]. Moreover, Rg3-induced apoptosis in A549 lung adenocarcinoma via downregulation of epidermal growth factor receptor and in human breast cancer (MDA-MB-231) cells, thus preventing breast cancer [9]. Furthermore, Rg3 has been reported to improve vascular function in spontaneously hypersensitive rats by increasing eNOS phosphorylation and inhibiting ICAM-1 expression, and act as an anticancerous agent by inhibiting the NF-*κ*B pathway in prostate cancer cells and colon cancer cells [10–14].

In our study, we explored the anti-inflammatory effects of Rg3-RGE* in vitro* and* in vivo*, using a lipopolysaccharide- (LPS-) induced septic shock mouse model. To the best of our knowledge, this is the first report to show that Rg3-RGE can potently inhibit LPS-induced septic shock in mice and inflammation in RAW 264.7 cells.

## 2. Materials and Methods

### 2.1. Reagents

The main reagents used in this study were as follows: Dulbecco's modified Eagle's medium (DMEM) (Daegu, Korea); fetal bovine serum (FBS) (WelGene Co., Korea); streptomycin and penicillin (Lonza, MD, USA); TRIZOL® reagent (Invitrogen, Carlsbad, CA, USA); oligodT, iNOS, COX-2, TNF-*α*, and IL-6 and IL-1*β* primers (Bioneer, Daejeon Korea); LPS (Escherichia coli 055:B5) and 3-(4,5-dimethylthiazol-2-yl)-2,5-diphenyltetrazolium bromide (MTT) (Sigma, St. Louis MO, USA). Specific antibodies used against phospho- and/or total form of ERK, JNK, p38, IKK *α*/*β*, I*κ*B*α*, NF*κ*B p65, iNOS, COX-2, and *β*-actin, and a rabbit HRP-conjugated secondary antibody were purchased from Cell Signaling Technology (Danvers, MA, USA). All other reagents and chemicals were obtained from Sigma Aldrich (St. Louis MO, USA).

### 2.2. Sample Preparation

**Figure 1:**
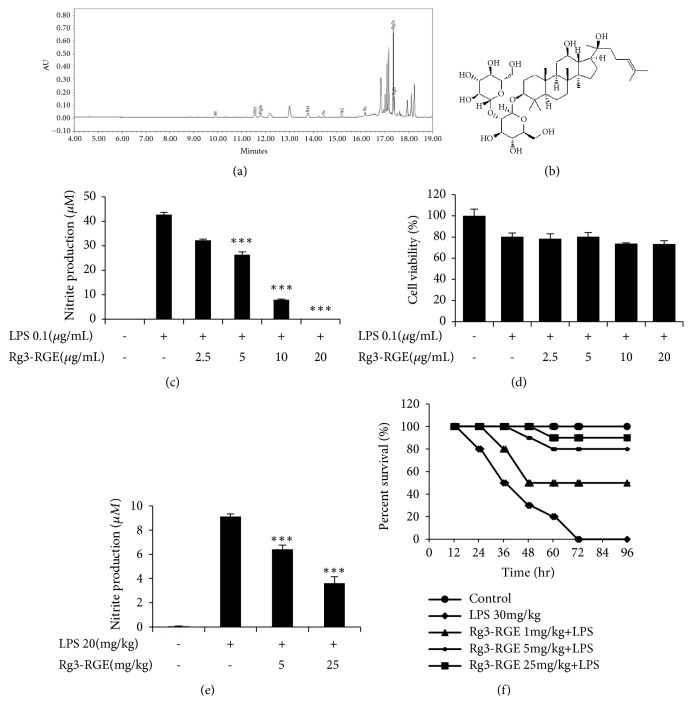
Inhibition of NO production by the Rg3-RGE extract. (a) HPLC chromatogram for Rg3-RGE. (b) Structure of Rg3-RGE. (c) RAW 264.7 cells were preincubated with Rg3-RGE for 30 min and then stimulated with LPS for 18 h. Cell supernatant was then mixed with equal amounts of Griess reagent and NO production was measured. (d) Effects of Rg3-RGE on cell viability as measured by the MTT assay. (e) Inhibition of NO production as measured in the serum of septic shock mice by using a commercial kit. (f) Survival rate was measured in ICR mice given a lethal dose of LPS for 96 h. Values in the bar graph are means ± SEM of three independent experiments. *∗∗∗p* < 0.001 are considered significant compared to the LPS-only group.

Red ginseng (stem/root=75:25) was extracted with distilled water and then 55% ethanol. A concentrated extract was prepared with multiple extractions. Subsequently, the extract was then subjected to high performance liquid chromatography (HPLC). The resulting profile of constituents is given in Table 1 and the structure is presented in Figure 1(b), with Rg3 being the most abundant (Figure 1(a)).

### 2.3. Animal Experiments

Male ICR mice (6–8 weeks old; 26–29 g) were purchased from Charles River, Orient Biotechnology, Gyeonggi-do, South Korea. The mice were housed in a specific-pathogen-free barrier facility at 21 ± 2°C with a relative humidity of 60 ± 10% under a 12-h light and dark cycle. Feed and water were provided* ad libitum*. All animal care and experimental procedures were carried out in accordance with internationally accepted guidelines on the use of laboratory animals (IACUC) and the protocols were approved by the Animal Care Committee of the College of Veterinary Medicine, Kyungpook National University, Daegu, South Korea (permission number for animal experiments: 2015-0075). The mice were divided into 4 groups for a survival study with 30 mg/kg LPS (for each group n = 10) and for clinical studies with LPS 20 mg/kg (for each group n = 6). For survival and clinical studies, the Rg3-RGE extract was administered orally 3 days prior to the LPS* i.p* treatment. The grouping was as follows: Group 1: Basal (vehicle treatment), Group 2: LPS (30 mg/kg or 20 mg/kg), Group 3: Rg3-RGE (5 mg/kg + LPS), and Group 4: Rg3-RGE (25 mg/kg + LPS).

### 2.4. Cell Culture

The murine macrophage cell line, RAW 264.7, obtained from the American Type Culture Collection was cultured in DMEM supplemented with 8% FBS and 1% of 100 IU/mL penicillin and 100 *μ*g/mL streptomycin sulfate. The cells were incubated at 37°C in a humidified atmosphere with 5% CO_2_.

### 2.5. NO Assay

NO was measured via the Griess reaction assay. Briefly, RAW 264.7 cells were seeded in 96-well plates and incubated with or without LPS (0.1 *μ*g/mL) in the absence or presence of Rg3-RGE at indicated concentrations for 18 h. The cell culture supernatants (100 *μ*L) were mixed with the Griess reagent (0.2% naphthylethylenediamine dihydrochloride and 2% sulfanilamide in 5% phosphoric acid) in ddH_2_O at equal volumes and incubated for 5 min at room temperature. The absorbance in each well was then analyzed at 540 nm in a microplate reader (Versamax Microplate Reader, Molecular devices, CA, USA).

### 2.6. Cell Viability (MTT) Assay

To determine the cytotoxic effects of Rg3-RGE, cell viability assay was assessed using the 3-(4,5-dimethylthiazol-2-yl)-2,5-diphenyltetrazolium bromide reagent, which was added to the culture medium at a final concentration of 0.1 mg/mL. After 4 h of incubation at 37°C in 5% CO_2_, the resulting violet colored crystals were dissolved in 100 *μ*L per well of dimethyl sulfoxide (DMSO) and the absorbance was measured at 560 nm.

### 2.7. RNA Extraction and Quantitative Reverse Transcription Polymerase Chain Reaction (qRT-PCR)

RAW 264.7 cells were pretreated with or without Rg3-RGE at indicated concentrations for 30 min and then stimulated with LPS (0.1 *μ*g/mL) for 18 h. For the* in vivo* study, male ICR mice were orally administered Rg3-RGE extract for 3 days, and LPS was then administered via an intraperitoneal injection (*i.p*.) at 20 mg/kg; thereafter, liver and testis tissues were collected for RNA extraction. After 18 h, total RNA was extracted using TRIZOL® reagent following the manufacturer's instructions. RNA was then annealed with Oligo-dt for 10 min at 70°C, cooled for 5 min on ice, reverse transcribed using a reverse transcriptase premix (Bioneer Co, Daejeon, Korea) in a 20-*μ*L reaction mixture, and run for 90 min at 42.5°C using a thermal cycler (Biometra GmbH, Germany). The reactions were terminated at 95°C for 5 min to inactivate the reverse transcriptase. The RT-PCR was performed using aliquots of cDNA obtained from the aforementioned reaction, and the PCR products were electrophoresed on a 1% agarose gel. The gel was then stained with ethidium bromide and visualized using Eagle Eyes image analysis software (Stratagene, LA Jolla, CA). The intensity of the bands was normalized against the intensity of the corresponding GAPDH band. Primer sequences used for qRT-PCR are given in Table 2.

### 2.8. Western Blot Analysis

RAW264.7 cells were either treated with Rg3-RGE (2.5–20 *μ*g/mL) or left untreated, in the presence or absence of LPS (0.1*μ*g/mL). For the* in vivo* study, male ICR mice were orally administered the Rg3-RGE extract for 3 days, and LPS was then administered* i.p*. at 20 mg/kg; thereafter, lungs and testis tissues were collected for protein extraction. Cytosolic and nuclear proteins were extracted according to the NE-PER ® Nuclear and Cytosolic Extraction Reagents manufacturer's instructions (Thermo Scientific, Seoul, Korea). Proteins were then measured using PROMEASURE assay kit (PROPREP, iNtRON Biotechonology, Sangdaewon-Dong, Korea). They were then separated by SDS-PAGE on a 10% polyacrylamide gel and subsequently transferred onto a polyvinyl difluoride (PVDF) membrane (Millipore, Immobilion ®-P, Billerica MA, USA). Nonspecific binding on the PVDF membrane was minimized with a blocking buffer containing 5% nonfat milk and 0.1% Tween-20 in Tris-buffered saline (TBS). The membranes were then incubated with specific primary antibodies overnight at 4°C followed by a 1 h incubation period with an HRP-conjugated anti-rabbit secondary antibody (1:3000 in TBS + 0.1% Tween-20). Bound antibodies were visualized using enhanced chemiluminescence (ECL) (Supex, Daegu, Korea) and images were analyzed using ImageJ software. *β*-actin was used as the internal control.

### 2.9. TNF-*α*, NO, and MDA Assays

Plasma was harvested from septic shock mice and then subjected to assays using different commercially available kits [i.e., TNF-*α* ELISA (R&D systems, Minneapolis, USA), NO and MDA (Abcam, Seoul Korea)]. Procedures were performed as per the manufacturer's instructions.

### 2.10. Hematoxylin and Eosin (H&E) Staining

The lung and testis tissues were collected from euthanized mice, placed in 10% neutral buffered formalin, and then processed for basic H&E staining according to established protocols [15].

### 2.11. Statistical Analysis and Normalization

Data are presented as mean ± SEM. One-way ANOVA and Dunnett's test were applied for the statistical evaluation of the data. Statistical analysis software (SAS version 9.4) was used for determination of statistical significance. Differences with *∗∗∗p* < 0.001 were considered significant.

## 3. Results

### 3.1. Effects of Rg3-RGE on LPS-Induced Inflammation

In our study, we found that Rg3-RGE sharply attenuated the levels of NO in a dose-dependent manner in RAW 264.7 cells and plasma of septic shock mice as shown in Figures 1(c) and 1(e). Moreover, no cytotoxic effects of Rg3-RGE at the dosages used were observed (Figure 1(d)). The Rg3 level in the extract is shown in Figure 1(a) by HPLC and Rg3 structure is shown in Figure 1(b). Moreover, Rg3-RGE also rescued mice from LPS-induced septic shock (Figure 1(f)).

### 3.2. Effects of Rg3-RGE on the Expression of Proinflammatory Mediators and Cytokines In Vitro and In Vivo

**Figure 2:**
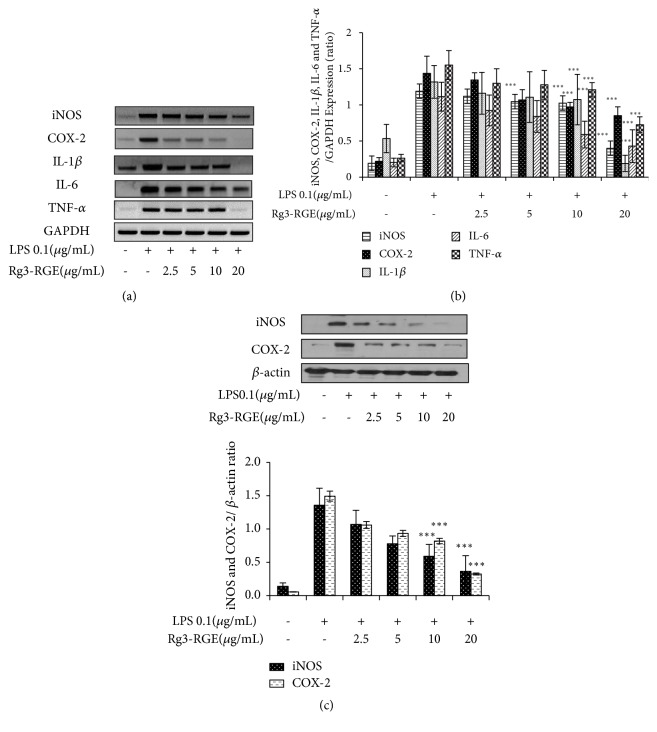
Suppression in the expression of proinflammatory mediators and cytokines by the Rg3-RGE extract. (a–b) RAW 264.7 cells were preincubated with Rg3-RGE for 30 min and then stimulated with LPS for 18 h. Later the expression of proinflammatory mediators and cytokines were determined by RT-PCR. Moreover in (c), proteins were extracted from RAW 264.7 cells and the level of iNOS and COX-2 were measured by western blotting. Values in the bar graphs are means ± SEM of three independent experiments. *∗∗∗p *< 0.001 are considered significant compared to the LPS-only group.

**Figure 3:**
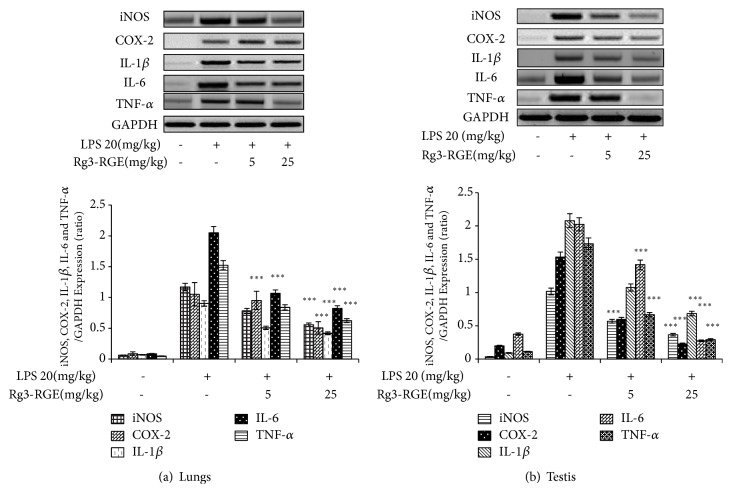
Suppression in the expression of proinflammatory mediators and cytokines in tissues by the Rg3-RGE extract. Total RNA from lungs (a) and testis tissue (b) were extracted and mRNA expression of iNOS, COX-2, IL-1*β*, IL-6, and TNF-*α* was determined by RT-PCR. GAPDH was used as an internal control. Image is representative of three independent experiments. Values in the bar graphs are means ± SEM of three independent experiments. *∗∗∗p *< 0.001 are considered significant compared to the LPS-only group.

**Figure 4:**
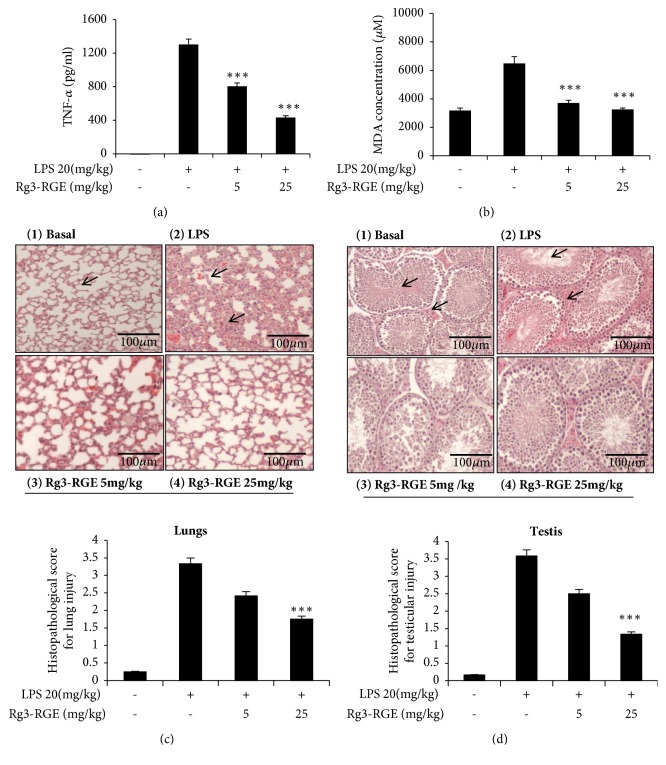
Reduction in serum TNF-*α* and MDA levels by Rg3-RGE. For the chronic study of septic shock in mice, using LPS, mice were treated orally with Rg3-RGE for 7 days and then were given an LPS injection. Three days later, they were euthanized and blood and tissues were collected to assess TNF-*α* and MDA levels using commercially available kits. (a) TNF-*α* levels and (b) MDA levels. Values in the bar graphs are means ± SEM of three independent experiments. *∗∗∗p* < 0.001 is considered significant compared to the LPS-only group. For hematoxylin and eosin staining, lung and testis tissues were stored in 10% neutral buffered formalin (NBF) and then stained according to a standardized protocol for H&E staining. ((c) (1-4)) Lungs and ((d) (1-4)) testis. Scale bar =100 *μ*m. For histopathological score, *∗∗∗p* < 0.001 are considered significant compared to the LPS-only group.

We analyzed whether Rg3-RGE can affect proinflammatory mediators and cytokine expression in RAW 264.7 cells. As shown in Figures 2(a)–2(c), Rg3-RGE not only suppressed the transcriptional and translational levels of iNOS, COX-2, interleukin (IL)-1*β*, IL-6, and TNF-*α* in RAW cells, but also reduced the mRNA levels for these proinflammatory cytokines in the lung and testis tissues of LPS-induced septic shock mice in a dose-dependent manner (Figures 3(a) and 3(b)). Moreover, the TNF-*α* level was also found to be dose-dependently reduced in the plasma obtained from the blood of septic mice, as shown in Figure 4(a).

Malondialdehyde (MDA) is a lipid peroxidation marker whose levels increase in response to oxidative stress [16]. LPS exposure causes increased production of oxygen free radicals and thus increases MDA levels. However, Rg3-RGE significantly diminished the levels of MDA in the plasma of LPS-treated mice, as shown in Figure 4(b). The results of the hematoxylin and eosin (H&E) staining of the lung and testis tissue indicated inhibition of inflammation in septic shock mice, as shown in Figures 4(c) and 4(d). A grading system was used to score for the alveolar and parenchymal histological changes (i.e., alveolar structure, inflammation, infiltration of alveolar septum with inflammatory cells, increased capillary permeability, hemorrhage, edema, and congestion). The grading system was scored, whether there is absence (0) or presence (1 for mild, 2 for moderate, 3 for marked, and score 4 for diffuse) in the alveolar tissues. The testicular damage was also scored from 0-4 with 0 having proper arrangement of seminiferous tubules with clear interstitial spaces, 1 having mild disruption of seminiferous tubules with little loss of morphology, 2 having the moderated damage of seminiferous tubules with loss of spermatids, disruption of Leydig cells, degeneration of germ cell layers, hemorrhage and edema in interstitium, 3 being the moderate to severe form of score 2 and 4 being the most severe form of score 3 with fibrosis and granuloma. This histological scoring was based on previous studies [17–20].

### 3.3. Signal Transduction of Rg3-RGE via the NF-*κ*B and MAPK Pathways

**Figure 5:**
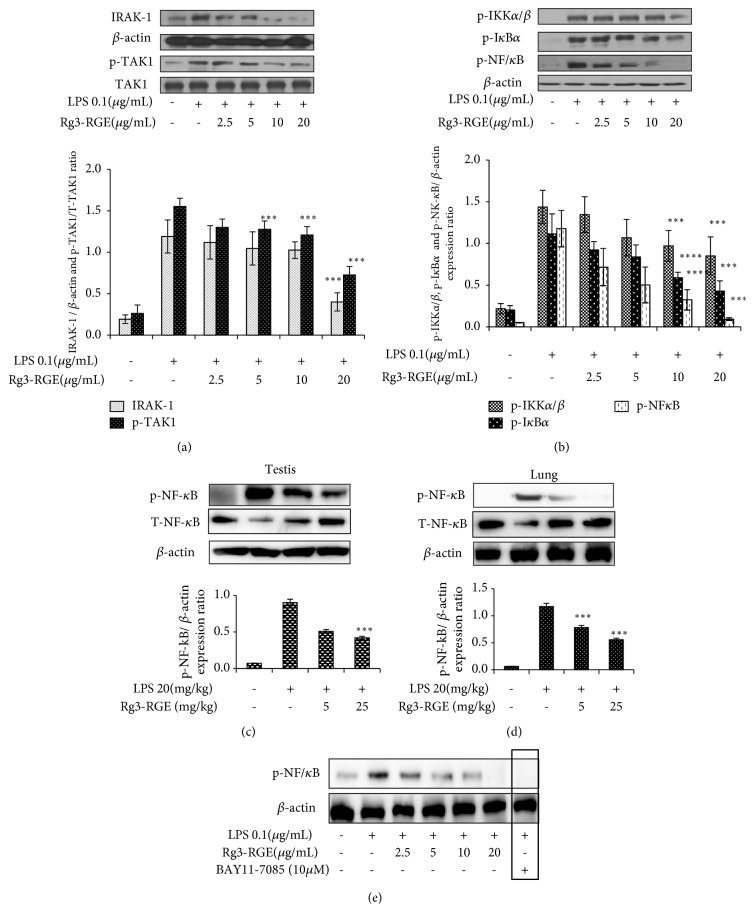
Rg3-RGE-induced signal transduction through the NF-*κ*B pathway and its inhibitor* in vitro* and* in vivo*. RAW 264.7 cells were preincubated with Rg3-RGE for 30 min and then stimulated with LPS for 18 h. Nuclear and cytosolic proteins were extracted from cells using the NE-PER® Nuclear and Cytosolic Extraction Reagents. *β*-actin was used as an internal control. Image is representative of three independent experiments. (a, b) Phosphorylation of NF-*κ*B pathway factors in RAW cells. (c, d) Inhibition of phosphorylation of p- NF-*κ*B in testis and lungs tissue. (e) Expression of BAY11-7085 (NF-*κ*B inhibitor). Values in bar graphs are means ± SEM of three independent experiments. *∗∗∗p* < 0.001 is considered significant compared to the LPS-only group.

In our study, Rg3-RGE* in vitro* in macrophages and* in vivo* in lungs and testis dose-dependently suppressed the phosphorylation of nuclear factor-kappa B (NF-*κ*B) and all its upstream factors (Figures 5(a)–5(d)). Moreover, BAY11-7058 which is NF-*κ*B pharmacological inhibitor was also inhibited by treatment of RAW cells with LPS confirming that Rg3-RGE strongly transduces its signal via NF-*κ*B pathway as shown in Figure 5(e).

**Figure 6:**
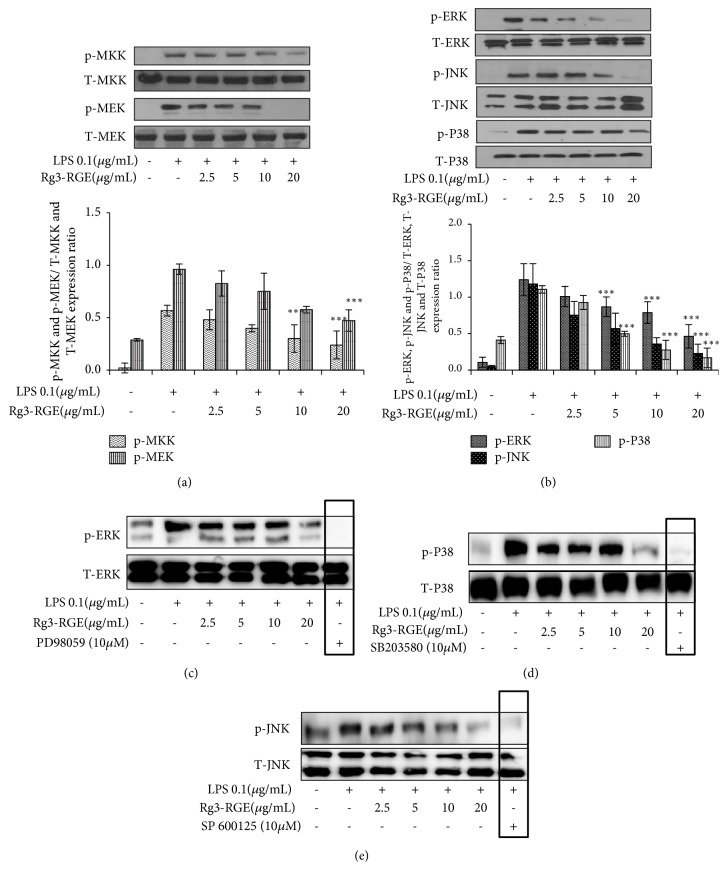
Rg3-RGE-induced signal transduction through the MAPK and its inhibitors in RAW 264.7 cells. RAW 264.7 cells were preincubated with Rg3-RGE for 30 min and then stimulated with LPS for 18 h. Nuclear and cytosolic proteins from testis and lungs were extracted using the NE-PER® Nuclear and Cytosolic Extraction Reagents. Total forms of respective factors were used as internal controls. Image is representative of three independent experiments. (a, b) Phosphorylation of MAPK pathway factors in RAW cells. (c-e) Expression levels of MAPK inhibitors. (c) PD 98059 (ERK inhibitor), (d) SB 203580(P38 inhibitor), and (e) SP 600125 (JNK inhibitor). Values in bar graphs are means ± SEM of three independent experiments. *∗∗∗p* < 0.001 are considered significant compared to the LPS-only group.

The mitogen-activated protein kinase pathway (MAPK) pathway is another important pathway for stress response in cells [21]. It is also costimulated with the NF-*κ*B pathway to elicit the inflammatory response. Similar to the NF-*κ*B pathway, it includes a variety of factors whose subsequent phosphorylation mediates the inflammatory response. Therefore, we assessed the effects of Rg3-RGE on the expression of factors involved in the MAPK pathway. As shown in Figures 6(a) and 6(b), Rg3-RGE dose-dependently inhibited the phosphorylation of both up- (MAPK Kinase (MKK) and MAPK extracellular signal-regulating kinase (MEK)) and downstream (Extracellular signal-Regulating Kinase (ERK), c-Jun N-terminal kinase (JNK), and p38 MAPK (P38)) factors of this pathway. For further confirmation, we checked the expression of Rg3-RGE in RAW 264.7 cells (Figures 6(c)–6(e)) by MAPK pathway factors specific inhibitors that had inhibited LPS expression potently in RAW 264.7 cells indicating that it is a potent anti-inflammatory agent exerting its effects via MAPK pathway as well.

## 4. Discussion

Natural products have been used for the treatment of almost all bodily ailments for centuries. However, given the scientific advancements, we are now able to investigate the molecular mechanisms underlying the effects of the compounds present in these natural products (herbs, shrubs, roots, leaves, and flowers). Ginseng, which is considered as panacea (especially in the Korean Peninsula), consists of a variety of compounds. Numerous studies have been conducted on ginseng extract as a whole and as single, isolated compounds [22, 23]. Even some of our previous research has also unraveled anti-inflammatory activities of a nonsaponin fraction called “Gintonin” in RAW 264.7 cells; however gintonin differs from Rg3-RGE in terms of composition and nature because it is a nonsaponin fraction from ginseng extract [24]. In addition to this, our recent* in vitro* research has also unraveled that Rg3-RGE mediated these anti-inflammatory effects via RXR*α*-PPAR*γ* heterodimeric nuclear receptors which was confirmed by the molecular docking analysis data [25]. Because there was no study available on the effects of Rg3-RGE especially in the* in vivo* sepsis studies, therefore we conducted this study to elucidate the complete anti-inflammatory mechanism of Rg3-RGE both* in vitro* and* in vivo*.

Nitric oxide (NO) is produced as a protective mechanism of cells towards foreign invasion. In general NO is a gaseous moiety that helps the cells to combat foreign invader however, if there is continuous persistence of foreign invader, NO is also continuously produced that serves detrimental to neighboring cells [26]. Therefore its timely regulation is the major mechanism of action for most of the anti-inflammatory drugs from allopathic or herbal origin. In our study we have shown that the Rg3-RGE potently suppressed the levels of nitric oxide both in the RAW cells that were stimulated* in vitro* with LPS and in mice that were given septic shock via LPS intraperitoneally.

Severe inflammation and sepsis have always been, and continue to be, major concerns for patients and doctors alike. A common cause of inflammation is infection by a bacteria or virus. Upon infection, proinflammatory molecules, cytokines and anti-inflammatory factors are released [27]. It is the balance between the pro- and anti-inflammatory agents that rids the body of the invading pathogen and protects it from the damage caused by inflammation. NO production is typically regarded as a mode of defense in cells against endotoxic shock, such as that induced by LPS [26]. The binding of LPS with Toll-like receptor 4 (TLR4) activates the downstream inflammatory signaling pathways via the production of proinflammatory mediators such as inducible nitric oxide synthase (iNOS) and cyclooxygenase-2 (COX-2) [28]. These proinflammatory mediators in turn activate the cascaded of proinflammatory cytokines like IL-1*β*, IL-6 and TNF-*α*. Primarily these three afore mentioned cytokines are deleterious to cells if there secretion is not combated properly [29, 30]. And according to our results, Rg3-RGE suppressed both the transcriptional and translational levels of these proinflammatory cytokines and mediators in both RAW cells and septic shock induced mice lungs and testis. Moreover MDA, which is produced as a result of lipid peroxidation by reactive oxygen species and which also serves to flare up inflammation and its levels are elevated in certain inflammatory diseases [31, 32] was also significantly reduced by Rg3-RGE.

Further going into mechanistics, in our study we have shown that Rg3-RGE exerted its anti-inflammatory effects via NF-*κ*B pathway. NF-*κ*B is one of the key pathways in inflammatory signaling. It is responsible for the induction of expression of an extensive range of transcription factors and genes that are linked to immunity, stress, and inflammation [33]. When the ligand-receptor (TLR4-LPS) union takes place, TGF-*β* activated kinase 1(TAK1) is activated via phosphorylation. Subsequently, phosphorylated TAK1 moves downstream to activate I*κ*B kinase (IKK*α*/*β*) by phosphorylation, which then phosphorylates and activates I*κ*B*α*. Phosphorylated I*κ*B*α* then sets NF-*κ*B free, whereby it translocates into the nucleus and initiates the transcription of inflammation-associated genes [34]. Besides, NF-*κ*B pathway, MAPK is also another pathway stress activated pathway. And similar to NF-*κ*B pathway it also consists of a serious of upstream (MKK and MEK) and downstream (ERK, JNK and P38) factors that are responsible for initiation of inflammation [21, 35]. And it is evident from our results that Rg3-RGE dose-dependently inhibited the phosphorylation of all up- and downstream factors of NF-*κ*B and MAPK pathway. Moreover we also counter confirmed the suppression of MAPK and NF-*κ*B pathways by using their specific inhibitors that strongly suppressed LPS production which proved clearly the Rg3-RGE's signal transduction by MAPK and NF-*κ*B pathways.

## 5. Conclusion

To the best of our knowledge, we are the first to report that Rg3-RGE's strong anti-inflammatory effects occur via the NF-*κ*B and MAPK pathways. This extract also potently inhibited the expression of NO, iNOS, COX-2, IL-1*β*, IL-6, and TNF-*α* at both the transcriptional and translational levels,* in vitro* and* in vivo.* Therefore, based on our findings we believe that prophylactic supplementation of Rg3-enriched red ginseng extract can be considered as good herbal remedy to combat inflammation in humans.

## Figures and Tables

**Table 1:** Profile of constituents present in the Rg3-RGE extract.

**Ginsenosides **	**Contents (mg/g)**
Rb1	3.86
20(S)-Rg3	44.91
Rc	1.20
Rb2	1.53
Rd	1.60
Rf	1.28
Rh1	3.71
20(S)-Rg2	3.55
20(R)-Rg3	6.78
Total	67.41

**Table 2:** Primer sequences for the polymerase chain reaction (PCR).

**Gene**	**Primer**	**Oligonucleotide sequence (5'-3')**
GAPDH	F	5'CAATGAATACGGCTACAGCAAC3'
	R	5'AGGGAGATGCTCAGTGTTGG3'
iNOS	F	5'CCCTTCCGAAGTTTCTGGCAGCAGC3'
	R	5'GGCTGTCAGAGCCTCGTGGCTTTGG3'
COX-2	F	5'-TCTCAGCACCCACCCGCTCA-3'
	R	5'-GCCCCGTAGACCCTGCTCGA-3'
IL-1*β*	F	5'CAGGGTGGGTGTGCCGTCTTTC3'
	R	5'TGCTTCCAAACCTTTGACCTGGGC3'
TNF-*ɑ*	F	5'TTGACCTCAGCGCTGAGTTG3'
	R	5'CCTGTAGCCCACGTCGTAGC3'
IL-6	F	5'-GTACTCCAGAAGACCAGAGG-3'
	R	5'-TGCTGGTGACAACCACGGCC-3'

## Data Availability

The data used to support the findings of this study are included within the article.

## Abbreviations

LPS: Lipopolysaccharides
NO: Nitric oxide
iNOS: Inducible NO synthase
TNF-*α*: Tumor necrotic factor alpha
COX-2: Cyclooxygenase-2
NF-*κ*B: Nuclear Factor kappa B
MAPK: Mitogen-activated protein kinases
TLR: Toll-like receptors
IL: Interleukin
ERK: Extracellular signal-regulated kinases
JNK: c-Jun N-terminal kinases
IKK: Inhibitor of kappa B kinase
I*κ*B/*α*: Inhibitor kappa B-alpha
DMEM: Dulbecco's modified Eagle's medium
FBS: Fetal bovine serum
MTT: 3-(4,5-Dimethylthiazol-2-yl)-2,5- diphenyltetrazolium bromide.
